# Neuroprotective role of bilirubin in Parkinson's disease

**DOI:** 10.3389/fnagi.2026.1768924

**Published:** 2026-03-23

**Authors:** Yifan Zhang, Hanwei Liu, Huaiwen Cao, Mingchen Deng, Zheng Han, Xinyu Wu, Qian Wan, Ruotong Wang, Yuanyuan Xu, Jingwei Lai, Yumeng Jiang, Lina Gong, Zhenqi Liu, Ke Lai, Haibo Shi

**Affiliations:** 1 Department of Otorhinolaryngology Head and Neck Surgery, Shanghai Sixth People's Hospital Affiliated to Shanghai Jiao Tong University, Shanghai, China; 2 Otolaryngology Institute of Shanghai Jiao Tong University, Shanghai, China

**Keywords:** bilirubin, dopaminergic neurons, inflammation, neurodevelopment, oxidative stress, Parkinson's disease

## Abstract

**Introduction:**

The progressive loss of dopaminergic neurons in the nigrostriatal pathway, driven by mechanisms such as oxidative stress, neuroinflammation, and ferroptosis, represents a hallmark of Parkinson's disease (PD). Notably, physiological or mildly elevated bilirubin levels demonstrate potent antioxidant and anti-inflammatory properties. This positions bilirubin as a compelling endogenous molecule with potential neuroprotective significance in PD. Furthermore, emerging evidence links early embryonic neurodevelopmental impairments to long-term PD risk, revealing a new dimension for protective interventions.

**Methods:**

This review synthesizes current evidence on the protective roles of bilirubin against PD, detailing its mechanisms in countering oxidative stress, modulating neuroinflammation, inhibiting ferroptosis, and supporting normal development of nigrostriatal dopaminergic circuits. Key molecular pathways—including Nrf2 activation, microglial polarization, and developmental signaling pathways such as Wnt/β-catenin and Shh—are critically examined.

**Results:**

Our analysis demonstrates that bilirubin directly neutralizes reactive species, preserves mitochondrial integrity, and promotes an anti-inflammatory milieu by inducing M2 microglia and modulating T-cell populations. Bilirubin also mitigates dopaminergic neuron injury by reducing iron deposition and activating the Nrf2 pathway. Beyond these classical mechanisms, bilirubin may fundamentally shape PD risk by orchestrating early embryonic development of dopaminergic neurons through key morphogenic signals, thereby ensuring robust neural circuit formation.

**Discussion:**

This review explores the multifaceted potential of bilirubin, framing it not only as a neuroprotectant against established PD pathologies but also as a developmental modulator. By integrating insights from neural development and classical neurodegeneration, this work will inspire future translational research into bilirubin-based therapeutic strategies to prevent or modify the progression of PD.

## 1 Introduction

Bilirubin (BL) is traditionally considered neurotoxic, however, contrary to this historical characterization, BL exhibits potent antioxidant and anti-inflammatory activity. Notably, neuroprotective effects are potentially conferred by both physiological and moderately elevated concentrations. A single molecule of BL can neutralize the neurotoxic effect induced by hydrogen peroxide (H_2_O_2_) at concentrations 1,500–3,000 times higher, thereby mitigating oxidative stress (Doré et al., 2000). BL also modulates CD8^+^ T cell activity and promotes regulatory T (Treg) cell proliferation, contributing to the suppression of inflammatory responses (Wagner et al., 2015). In autoimmune encephalitis and sepsis, BL can mitigate tissue damage by modulating T-cell activity and population dynamics (Kim et al., 2021; Tran et al., 2020). Notably, BL selectively engages PPARα–a receptor expressed in neurons and glial cells that governs neurogenesis during differentiation to provide neuroprotection (Fidaleo et al., 2014; Gordon et al., 2020). In addition, individuals with Gilbert's syndrome (defined by asymptomatic serum bilirubin elevations >17.1 μmol/L) show lower incidence of cardiovascular and neurodegenerative disorders than the general population (Vítek and Tiribelli, 2023). Collectively, these findings suggest that physiological or mildly elevated BL levels may confer protection against a range of diseases, including Parkinson's disease (PD).

PD is a neurodegenerative disorder characterized by core motor symptoms—including resting tremor, dystonia, and gait disturbances—frequently accompanied by non-motor manifestations such as dementia, sleep disorders, and autonomic dysfunction (Volta et al., 2015). The pathogenesis of PD is multifactorial, with the prevailing hypothesis implicating aberrant accumulation of α-synuclein (α-Syn) in dopaminergic (DA) neurons of the substantia nigra pars compacta within the basal ganglia. This pathological process drives neuronal degeneration and impaires striatum dopamine transmission, ultimately disrupting motor and cognitive function (Calabresi et al., 2014; Volta et al., 2015). The nigrostriatal pathway is particularly vulnerable to oxidative stress, neuroinflammation, and genetic mutations (e.g., parkin) due to its high synaptic density, extensive unmyelinated axonal networks, and elevated metabolic demands (Bolam and Pissadaki, 2012). Increasing evidence suggests that neurodegenerative pathology may also originate from defects in embryonic development (Catlin and Schaner Tooley, 2024; Hegarty et al., 2013; Schwamborn, 2018), underscoring the critical role of ventral midbrain DA neuron development in elucidating PD mechanisms. Dysregulation of the critical developmental pathways—including sonic hedgehog (Shh) signaling (Echelard et al., 1993; Ho and Scott, 2002), Wnt signaling (Castelo-Branco et al., 2003, 2006), and the nuclear receptor Nurr1 (Zetterström et al., 1996)—during early neural maturation may latent susceptibility to PD. We aim to demonstrate that the antioxidant and anti-inflammatory properties of bilirubin can protect dopaminergic neurons and delay the onset of Parkinson's disease.

## 2 Protective effects of BL in PD

### 2.1 What does bilirubin mean for Parkinson's disease?

BL paradoxically exhibits neurotoxic and neuroprotective roles via antioxidant and anti-inflammatory activity that may modulate the pathophysiology of various central nervous system disorders (Vítek and Tiribelli, 2021). Although BL has been proposed as a potential biomarker for PD, existing clinical studies have reported conflicting results, reflecting the complex, and stage-dependent dynamics of oxidative stress in the brain.

On one hand, a robust body of evidence suggests that lower BL levels are associated with increased PD vulnerability. Epidemiological studies in the U.S. population indicate a negative correlation between serum BL levels and PD incidence, with maximal protective effects observed at 10.84 μmol/L (Su et al., 2024). This protective hypothesis is corroborated by multiple independent international cohorts. For instance, Italian and Japanese cohort studies have similarly demonstrated that lower serum bilirubin levels are significantly associated with a higher risk of developing PD and greater motor severity (Hatano et al., 2016; Su et al., 2024). Consistent with these findings, Chinese cohort studies have shown reduced indirect BL levels in PD patients (*p* = 0.015), with the most significant reductions observed in early-stage disease (*p* = 0.013; Qin et al., 2015). Subtype analyses indicate that reductions in indirect BL occur predominantly in the postural instability/gait difficulty subtype of PD (*p* < 0.001; Li et al., 2019).

Conversely, an opposing subset of literature reports elevated BL levels in specific PD subpopulations, likely representing a compensatory physiological response. Albillos et al. (2021) reported elevated BL levels in PD patients compared with controls, particularly in treated individuals with advanced-stage disease. Clinical staging analyses by independent research groups show that BL levels are higher in moderate PD (Hoehn and Yahr stage ≤ 3) compared with controls (0.56 ± 0.26 vs. 0.45 ± 0.22 mg/dL; *p* < 0.001; Macías-García et al., 2019; Songsomboon et al., 2020). Similarly, treatment-naïve PD cohorts exhibit elevated BL levels (*p* < 0.001) and an inverse correlation between BL and levodopa requirements (*p* = 0.012; Moccia et al., 2015). Neuroimaging studies using PET-CT further link BL levels to striatal dopamine transporter availability (*p* < 0.001), with the strongest association observed in the posterior putamen (*p* < 0.001; Lee et al., 2019). Meta-analytic findings synthesizing diverse clinical trials further support elevated total BL (*p* = 0.018) and direct BL (*p* = 0.008) in PD populations (Jin et al., 2020).

Current evidence indicates that BL levels are elevated in PD patients which progressively decline with disease duration and show an inverse correlation with levodopa equivalent daily dose. This pattern likely reflect compensatory upregulation of heme oxygenase-1 (HO-1) and subsequent BL production in response to nigral oxidative stress and neuroinflammation during the early PD stages (Cuadrado and Rojo, 2008; Schipper et al., 1998, 2009). Furthermore, the antioxidant and anti-inflammatory properties of BL may mitigate levodopa-induced free radical generation, potentially reducing therapeutic requirements in PD patients (Jayanti et al., 2021; Martignoni et al., 1999; Moccia et al., 2015; Scigliano et al., 1997).

### 2.2 Neuroprotective role of BL in PD

The mechanistic interplay between BL and PD has gained increasing attention, propelled by accumulating evidence of neuroprotective effects of BL and its potential utility as a PD biomarker. PD is defined by DA neuron degeneration, α-Syn aggregation, neuroinflammation, and oxidative stress. Through its dural antioxidant and anti-inflammatory actions, BL may directly target these key mechanisms underlying PD pathogenesis.

#### 2.2.1 Anti-oxidant effects

Oxidative stress in the nigrostriatal pathway is a defining pathological hallmark of PD, and BL, as an endogenous antioxidant, can exert neuroprotective effects by attenuating DA neuron degeneration (Jayanti et al., 2021; Kong et al., 2023).

Mitochondrial dysfunction plays a pivotal role in PD-associated oxidative pathology through four interconnected mechanisms: progressive depletion of nigral glutathione (GSH; Pearce et al., 1997); impaired mitophagy resulting from LRRK2, PINK1, or PRKN mutations (Eldeeb et al., 2022; Wang et al., 2022); α-Syn oligomer-induced disruption of mitochondrial membranes leading to electron transport chain dysfunction (Burtscher et al., 2021); and inhibition of mitochondrial complex I by neurotoxins or intrinsic defects, ultimately amplifying reactive oxygen species (ROS) generation (Mani et al., 2021).

BL endowed with high-affinity binding to ROS, demonstrating exceptional efficiency in scavenging oxidizing agents (Thomas et al., 2022). Evidence from Mo/Hu APP695^swe^ transgenic mice supports this antioxidant capacity, as reduced neuronal BL levels in these models are accompanied by heightened susceptibility to H_2_O_2_-induced neurotoxicity (Takahashi et al., 2000). Mechanistically, the tetrapyrrole structure of BL enables redox cycling via its reactive C-10 bridge, facilitating the neutralization of superoxide anions (${\text{O}}_{2}^{-}$) and hydroperoxide radicals (${\text{HO}}_{2}^{-}$; Farrera et al., 1994). Notably, BL shows superior antioxidant potency compared with other endogenous compounds, as reflected by its lower IC50 values in superoxide decomposition assays and its enhanced peroxyl radical scavenging activity in unsaturated fatty acid systems (Cui et al., 2023; Farrera et al., 1994).

Reactive nitrogen species (RNS), particularly nitric oxide (NO), are generated via the reduction of nNOS, inducible NO synthase (iNOS), nitrate, and nitrite, and serve as key mediators of oxidative stress (Stykel and Ryan, 2022). In PD, nNOS expression is upregulated in the substantia nigra (Eve et al., 1998), whereas microglial iNOS levels are elevated across multiple neurodegenerative disorders (Lowenstein and Padalko, 2004). Excessive calcium-dependent NO production impairs mitochondrial function in DA neurons, thereby disrupting synaptic transmission, vesicular trafficking, and dopamine homeostasis (Giesen et al., 2020). Furthermore, α-Syn undergoes RNS-mediated nitration in PD brains, promoting dimer formation that accelerates oligomerization kinetics (Burai et al., 2015). Clinically, elevated serum RNS and nitrated α-Syn correlate with accelerated disease progression and poorer motor performance in PD patients (Vicente Miranda et al., 2017).

**Figure 1:**
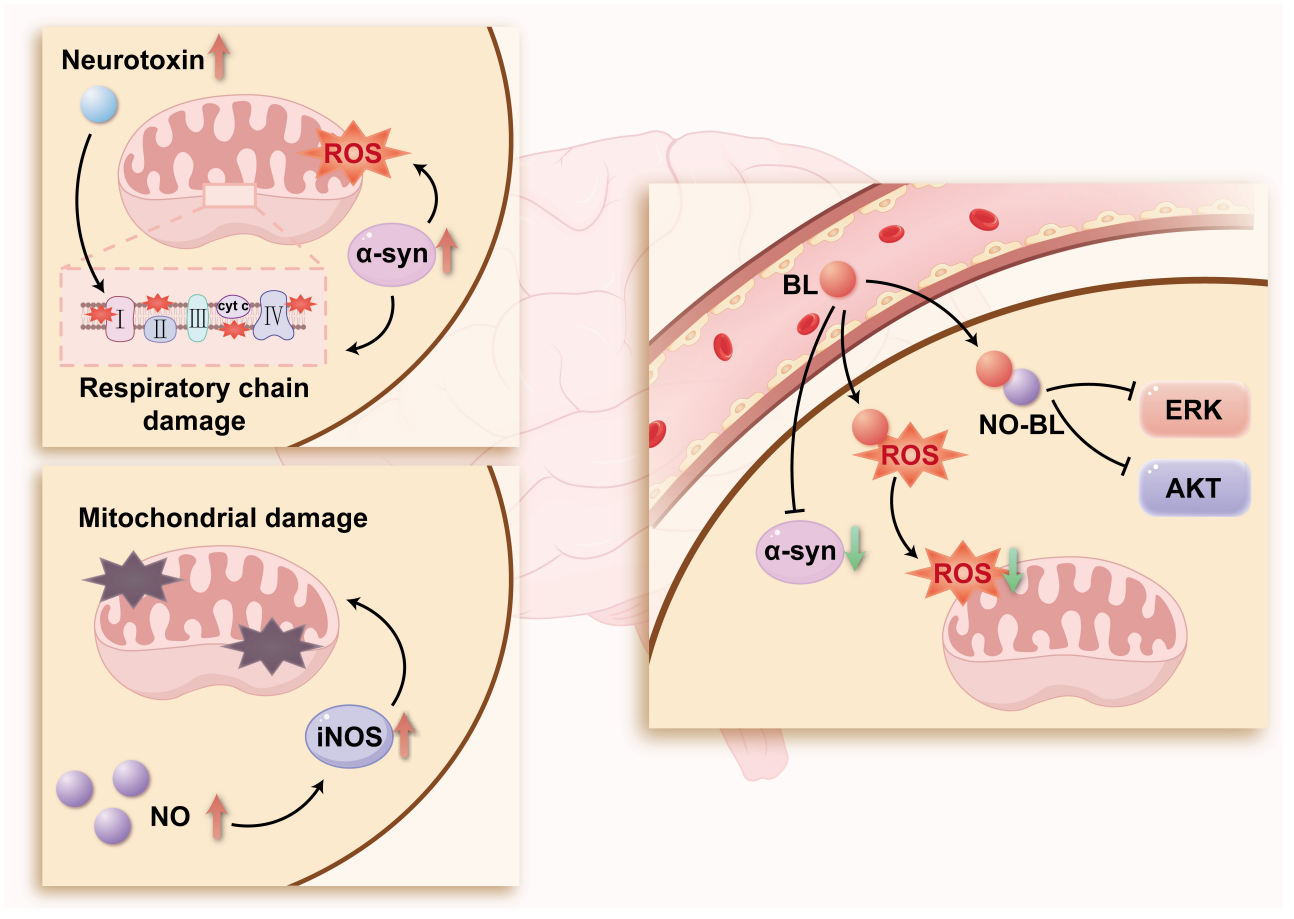
Effects of bilirubin on oxidative stress in DA neurons (NO, nitric oxide; BL, bilirubin; AKT, AKT serine kinase; ROS, reactive oxygen species; ERK, extracellular signal-regulated kinase; iNOS, inducible NO synthase).

BL can neutralize both extracellular and intracellular nitrosation reactions (Mancuso et al., 2003). NO scavenged by BL to generate BL-NO, an N-nitrosylated derivative proposed as a novel biomarker of oxidative and nitrosative stress. Neuroprotective effects of BL have also been proved under conditions of neurotrophic factor deprivation (Barone et al., 2009). In this context, NO activates a signaling cascade that elevates extracellular signal-regulated kinase (ERK) activity beyond baseline levels, partially preventing cell death by inhibiting phosphorylation of downstream effectors, including Akt/protein kinase B and ERK1/2 (Hansen et al., 2020; Mancuso et al., 2008). Through these mechanisms, BL may mitigate nitrative stress in DA neurons by neutralizing excess NO, thereby potentially reducing both neuronal loss and pathological α-Syn aggregation (Figure 1).

#### 2.2.2 Attenuation of inflammatory response

Neuroinflammation is a key pathological mechanism in neurodegenerative diseases and has been strongly implicated in PD. In 1988, McGeer et al. (1988) and (Tansey et al., 2022) identified HLA-DR? reactive microglia in postmortem PD brains, establishing one of the earliest links between neuroinflammation and PD. Epidemiological data further support this connection: patients with inflammatory bowel disease exhibit a 28%−30% increased risk of developing PD (Zhu et al., 2019), and Epstein-Barr virus infection has been linked to PD-like symptoms, highlighting the potential role of immune dysregulation in PD pathogenesis (Espay and Henderson, 2011). Postmortem analyses have confirmed astrocyte activation in the substantia nigra of PD patients, accompanied by elevated pro-inflammatory cytokines [tumor necrosis factor-α (TNF-α), IL-1β, transforming growth factor-β, and IL-6], ROS, NO, pro-apoptotic proteins, and T-cell infiltration. Collectively, these findings reinforce the concept that neuroinflammation is a fundamental driver of PD progression (Rajan et al., 2023; Tansey et al., 2022; Xu et al., 2023).

BL has been recently considered as an endogenous anti-inflammatory factor with systemic effects across the cardiovascular, digestive, and nervous systems. Epidemiological studies show that low total BL levels (near or below the physiological lower limit of 0.4–1 mg/dL) are associated with an increased risk of neurological disorders (Creeden et al., 2021; Gazzin et al., 2016; Jayanti et al., 2023; Vítek and Tiribelli, 2021), whereas physiological concentrations (0.1–1.2 mg/dL) inhibit NF-κB signaling and mitigate lipopolysaccharide (LPS)-induced sepsis in murine models (Li et al., 2020). The anti-inflammatory properties of BL are further supported by findings in experimental autoimmune encephalomyelitis (Liu et al., 2008). Mechanistically, BL suppresses iNOS expression, stimulates prostaglandin E_2_ production, impedes leukocyte migration, and attenuates Toll-like receptor-4 (TLR4)-mediated iNOS upregulation and interferon-β (IFN-β) secretion (Idelman et al., 2015; Wei et al., 2015).

Accumulating evidence indicates that BL exerts neuroprotective effects in neurodegenerative diseases, with PD serving as a prototypical example. Although elevated BL are well known for its neurotoxic role, physiological concentration appears to confer protection against neuroinflammation (Jayanti et al., 2021, 2023; Mancuso, 2017). Moderate physiological BL concentrations preserve blood-brain barrier (BBB) integrity primarily through potent antioxidant and anti-inflammatory mechanisms. By scavenging cerebral oxygen free radicals and reducing TNF-α production, BL mitigates direct oxidative, and inflammatory damage to the BBB. Consequently, this structural preservation effectively limits the infiltration of peripheral immune cells, such as T-cells and neutrophils, into the brain parenchyma, thereby significantly attenuating downstream neuroinflammatory responses (Brito et al., 2014; Calabrese et al., 2004; Jayanti et al., 2022; Liu et al., 2003). Building on these findings, BL-based therapeutics, including heme-derived nanoparticles, are currently under investigation for their capacity to modulate neuroinflammation and oxidative stress in neurodegenerative disorders (Yin et al., 2022).

##### 2.2.2.1 BL modulates innate immunity in PD

The innate immune system plays a central role in the pathogenesis of PD (Tansey et al., 2022). Microglia and astrocytes, along with cellular pattern recognition receptors, constitute the core components of the innate immune response in PD (Gustavson and Miyake, 2016; Rajan et al., 2023). Among pattern recognition receptors, TLRs and NOD-like receptors (NLRs) are particularly critical, as they activate mitogen-activated protein kinase (MAPK) and NF-κB signaling pathways through receptor proteins or associated kinases (Wallings and Tansey, 2019). These receptors can also assemble inflammasome complexes, such as NLR family pyrin domain containing 3 (NLRP3), in coordination with absent in melanoma 2 (AIM2), resulting in the release of pro-inflammatory cytokines—including interleukin-1β (IL-1β), IL-18, and TNF-α–and thereby amplifying neuroinflammation (Elinav et al., 2011; Harms et al., 2021; Sandhir et al., 2017).

Notably, TLR4 ablation impairs microglial phagocytosis of α-Syn, facilitating its pathological accumulation (Stefanova et al., 2011). This accumulation further drives microglial polarization toward the pro-inflammatory M1 phenotype. Although M2-polarized microglia exert neuroprotective effects, sustained M1 activation or impaired transition to the M2 phenotype contributes to neurodegeneration (Subramaniam and Federoff, 2017). The entry of α-Syn into microglia is mediated by Fyn kinase, which also regulates the microglial potassium channel Kv1.3. Upregulation of Kv1.3 promotes M1 polarization (Rajan et al., 2023). Additionally, microRNA dysregulation—particularly miR-155 overexpression—has been shown to exacerbate M1 polarization and its detrimental effects (Thome et al., 2016).

In PD, elevated intracellular free Fe^2+^ leads to microglial iron overload, driving excessive ROS production, increased release of IL-1β and TNF-α, and enhanced α-Syn aggregation (Yu et al., 2023). Astrocytes, the most abundant glial cell type in the CNS, are similarly implicated in PD pathology. Activated microglia secrete IL-1α, TNF-α, and complement component C1q, which induce the transformation of astrocytes into the neurotoxic A1 phenotype (Liddelow et al., 2017; Phatnani and Maniatis, 2015). Postmortem analyses of PD brains show marked upregulation of complement component C3 and increased numbers of A1 astrocytes, paralleling observations in other neurodegenerative disorders (Liddelow et al., 2017).

Emerging evidence suggests that BL can abnormally activate TRPM2 channels, leading to pathological calcium influx and microglial overactivation. Conversely, physiological BL levels appear to exert regulatory effects that may modulate microglial function (Liu et al., 2023). Modulating microglial polarization—by suppressing pro-inflammatory M1 phenotypes or promoting neuroprotective M2 states is increasingly recognized as a strategy to mitigate neuroinflammation in PD, wherein, BL may play a role in facilitating this phenotypic shift (Yin et al., 2022).

PPARs are key regulators of microglial function (Agarwal et al., 2017; Subramaniam and Federoff, 2017). PPAR agonists have demonstrated neuroprotective effects across multiple neurodegenerative disorders, including PD (Agarwal et al., 2017). In MPTP-induced PD models, PPAR activation preserves DA neurons in the substantia nigra pars compacta and maintains striatal axon integrity (Pisanu et al., 2014). Specifically, PPARα activation reduces LPS-induced IL-1β production (Subramaniam and Federoff, 2017), whereas rosiglitazone-mediated PPARγ activation decreases TNF-α secretion (Pisanu et al., 2014). Together, these mechanisms mitigate M1 microglial polarization, promote M2 transition, and prevent degeneration of tyrosine hydroxylase (TH)-positive neurons in the nigrostriatal pathway (Carta et al., 2011; Dehmer et al., 2004). Notably, BL functions as a direct PPARα ligand (EC50 = 9.0 μM), enhancing transcriptional activity via corepressor-to-coactivator exchange (Gordon et al., 2019, 2020). This facilitates PPAR response element (PPRE) occupancy in target gene promoters and downregulates fatty acid synthase (FAS) gene expression in hepatocytes (Creeden et al., 2021). Collectively, these findings support a mechanism in which BL may attenuate PD-associated neuroinflammation through dual PPAR modulation: acting as a PPARα agonist and enhancing PPARγ expression. Such coordinated actions would suppress M1 microglial activation, reduce pro-inflammatory cytokine release (particularly TNF-α), promote M2 polarization, and ultimately protect nigrostriatal DA neurons from inflammatory injury.

**Figure 2:**
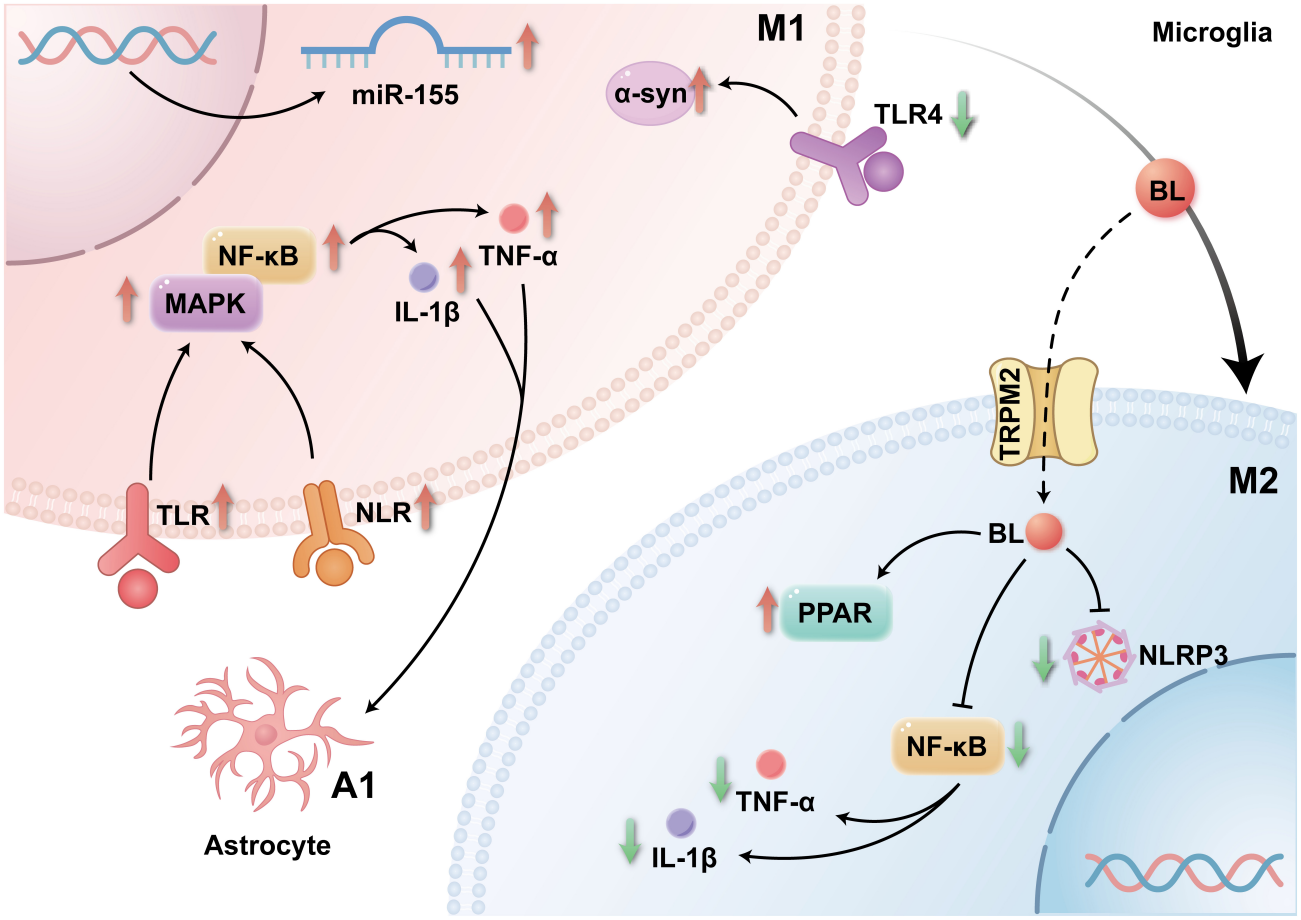
Bilirubin alleviates neuroinflammation through innate immune inhibition (NF-κB, nuclear factor kappa-light-chain-enhancer of activated B cells; MAPK, mitogen-activated protein kinase; TLR, toll-like receptor; NLR, NOD-like receptor; TRPM2, transient receptor potential melastatin 2; PPAR, peroxisome proliferator-activated receptor; NLRP3, NOD-like receptor family pyrin domain containing protein 3; TNF-α, tumor necrosis factor-alpha; IL-1β, interleukin-1 beta).

Emerging evidence suggests that BL may exert neuroprotective effects in PD by modulating NF-κB signaling and inflammasome activity (Li et al., 2020). Experimental studies using heme-derived nanoparticles show that BL, generated through heme catabolism alongside carbon monoxide (CO), suppresses nuclear translocation of the NF-κB p65 subunit in BV2 microglial cells polarized to the M1 phenotype. This effect is accompanied by a marked increase in M2 microglial populations (91.3% vs. 1.11% at baseline), as indicated by upregulated arginase-1 (Arg-1) expression (Yin et al., 2022). Mechanistic studies further demonstrate that BL pretreatment significantly attenuates LPS-induced NLRP3 inflammasome activation in RAW 264.7 macrophages, inhibiting both IL-1β expression and phospho-p65 nuclear translocation (Lin et al., 2019). *In vivo* findings corroborate these results: BL administration in murine peritonitis models reduces myeloperoxidase activity, suppresses secretion of pro-inflammatory cytokines (IL-1β, TNF-α, and IL-6), downregulates NLRP3 and IL-1β mRNA expression in peritoneal exudate cells, and decreases protein levels of phospho-p65 and mature IL-1β (Lin et al., 2019). Taken together, these observations support a model in which BL mitigates PD-associated neurodegeneration through multimodal anti-inflammatory mechanisms, including inhibition of NF-κB nuclear translocation, downregulation of p65 signaling, suppression of NLRP3 inflammasome assembly, and reduction of pro-inflammatory cytokine production. These coordinated actions likely contribute to the preservation of neuronal viability in PD pathogenesis (Figure 2).

##### 2.2.2.2 BL regulates adaptive immunity in PD

Postmortem analyses of PD patients and animal models consistently reveal significant blood-brain barrier disruption, accompanied by enhanced peripheral immune cell migration and prominent T cell infiltration (predominantly CD3^+^, CD4^+^, and CD8^+^ subsets) within the substantia nigra (Tansey et al., 2022; Xu et al., 2023). Peripheral CD4^+^ T cell populations are reduced, whereas the suppressive activity of CD4^+^FOXP3^+^ Tregs is augmented (Brochard et al., 2009; Rosenkranz et al., 2007). Sulzer et al. (2017); Appel (2009); Li et al. (2021). demonstrated that CD4^+^ T cells in PD patients specifically recognize α-Syn epitopes, triggering FAS receptor-mediated apoptotic signaling and increased secretion of inflammatory mediators, including IL-10. CD4^+^ T cells exhibit the capacity to differentiate into both pro-inflammatory [T helper-1 (TH1) and TH17] and anti-inflammatory (TH2 and Treg) subsets (Li et al., 2021; Xu et al., 2023). During early PD pathogenesis, activation of both TH1 and Treg populations is elevated; however, with disease progression, the proportion of pro-inflammatory cells increases whereas Treg-mediated neuroprotective effects are attenuated. This imbalance exacerbates cerebral immune dysregulation, ultimately accelerating PD progression (Baird et al., 2019; Williams et al., 2021).

Emerging evidence suggests that α-Syn and microglia act synergistically to exacerbate T cell–mediated neuroinflammation in the central nervous system (Sulzer et al., 2017). Nitrated α-Syn stimulates pro-inflammatory cytokine release from T cells while suppressing Treg function (Benner et al., 2008; Reynolds et al., 2010). A reciprocal activation loop exists between microglia and T cells (Xu et al., 2023): M1-polarized microglia secrete IL-6 and IL-1β which, in the presence of transforming growth factor-β, promote Th17 differentiation while inhibiting Treg development in naïve T cells (Zhang C. et al., 2018; Zhou et al., 2007). Additionally, microglia-derived chemokines (e.g., CXCL9) engage CXCR3 receptors on CD8^+^ T cells and Th1 cells, enhancing TNF-α production (Cui et al., 2020; Turner et al., 2007). Conversely, Th1-derived IFN-γ and TNF-α activate NF-κB signaling, leading to upregulation of TLR4 and myeloid differentiation primary response 88 (MyD88) expression (Bosisio et al., 2002). This cascade expands the population of neurotoxic microglia and amplifies the release of pro-inflammatory mediators (TNF-α, IL-1β, and IL-6), thereby perpetuating the neuroinflammatory cycle (Zeng et al., 2012).

BL has emerged as an immunomodulatory factor influencing adaptive immune responses. Transplantation studies demonstrate that BL treatment significantly reduces serum IFN-γ levels while promoting Treg expansion (Pareek et al., 2022), suggesting a dual mechanism that suppresses both neurotoxic microglial activation and effector T cell responses, while enhancing Treg-mediated protection of DA neurons. Complementary evidence from lung cancer models shows that BL nanoparticles (BRNPs) induce apoptotic pathways and promote Treg differentiation in co-culture systems, while simultaneously counteracting cigarette smoke extract-induced anti-apoptotic effects and Th17 polarization (Zhu et al., 2025). The anti-inflammatory potential of BL has also been validated in neurodegenerative disease models: in experimental autoimmune encephalomyelitis, BRNPs attenuate antigen-presenting cell maturation by scavenging ROS generated during dendritic cell and macrophage antigen uptake, thereby inhibiting naïve CD4^+^ T cell differentiation into Th17 cells and reducing pro-inflammatory Th17 populations (Kim et al., 2021).

Collectively, these findings support a model in which BL exerts neuroprotective effects in PD through coordinated modulation of adaptive immunity. Mechanistically, BL-mediated protection involves downregulation of IFN-γ production, attenuation of cytotoxic microglial activation and CD4^+^ T cell responses, expansion of Treg populations, and suppression of Th17 cell differentiation. This multifaceted immunomodulation shifts the immune balance from pro-inflammatory to anti-inflammatory states, thereby reducing neuroinflammation and preserving neuronal integrity in PD pathogenesis.

#### 2.2.3 Nuclear factor erythroid 2–related factor 2 (Nrf2) activation

Nrf2, a ubiquitously expressed transcription factor, functions as a master regulator of cellular responses to oxidative stress (Fão et al., 2019). Under basal conditions, Nrf2 is sequestered in the cytoplasm by Kelch-like ECH-associated protein 1 (Keap1). During oxidative stress, characterized by excessive ROS, Nrf2 dissociates from Keap1 and translocates to the nucleus (Dinkova-Kostova and Abramov, 2015; Sies and Jones, 2020). There, it binds to antioxidant response elements to drive the transcription of cytoprotective genes (Itoh et al., 1997, 1999). This Nrf2-mediated pathway reduces cellular oxidative damage, mitigates mitochondrial lipid peroxidation, and upregulates key antioxidant enzymes (Dinkova-Kostova and Abramov, 2015; Sies and Jones, 2020).

In PD, the substantia nigra DA neurons engaged marked Nrf2 upregulation with enhanced nuclear accumulation (Fão et al., 2019; Yamazaki et al., 2015). This compensatory response is supported by evidence that Nrf2-deficient mice display severe DA neuron loss and exacerbated microglia-mediated neuroinflammation (Rojo et al., 2010). Paradoxically, several pathways implicated in PD pathogenesis—including glycogen synthase kinase-3β (GSK-3β), p38 MAPK, and nuclear factor-κB (NF-κB)—are upregulated and inhibit Nrf2 activity (Alural et al., 2015; Mogi et al., 2007; Yasuda et al., 2011). Simultaneously, the phosphoinositide 3-kinase (PI3K)/Akt pathway, which positively regulates Nrf2, is significantly attenuated in PD. These alterations collectively impair Nrf2-driven antioxidant and anti-inflammatory responses, resulting in diminished expression of downstream protective enzymes such as HO-1 and GSH (de Oliveira et al., 2016).

BL has been shown to promote nuclear translocation of Nrf2, thereby upregulating HO-1 and enhancing the production of key antioxidant enzymes, including GSH, superoxide dismutase (SOD), and NAD(P)H quinone oxidoreductase 1 (NQO1) (Alural et al., 2015). In lymphatic endothelial cell models, pretreatment with 20 μM BL significantly reduces nuclear NF-κB p65 and total iNOS expression following H_2_O_2_ exposure, compared with controls (Huang et al., 2022). Collectively, these findings suggest that BL exerts cytoprotective effects against oxidative stress–induced apoptosis through two complementary mechanisms: preservation of intracellular redox homeostasis and coordinated regulation of the pro-inflammatory NF-κB/iNOS pathway alongside the cytoprotective Nrf2/HO-1 signaling axis (Huang et al., 2022; Zhao et al., 2024).

**Figure 3:**
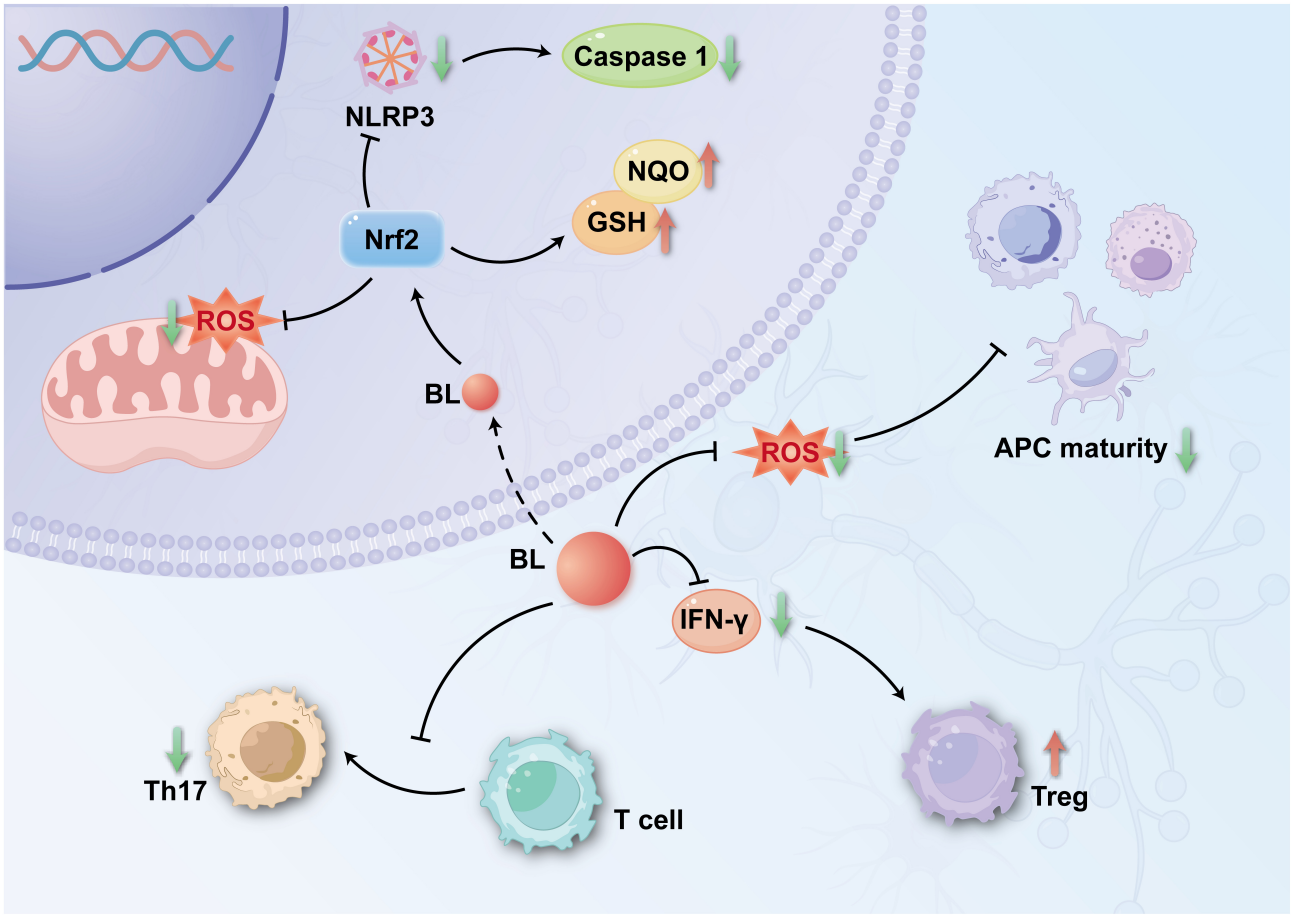
Bilirubin attenuates neuroinflammatory injury via regulation of T-cell differentiation and Nrf2 signaling (Nrf2: nuclear factor erythroid 2-related factor 2; NQO, NAD(P)H:quinone oxidoreductase; GSH, glutathione; IFN-γ, interferon-gamma).

In PD, activation of Nrf2 exerts neuroprotective effects by counteracting neuroinflammation. Activated Nrf2 orchestrates a multifaceted cytoprotective response through four principal mechanisms: scavenging ROS, downregulating NLRP3 expression, suppressing caspase-1 cleavage, and inducing NQO1 (Afonina et al., 2017; Hennig et al., 2018; Ji et al., 2020). In parallel, Nrf2 activation promotes p62-dependent mitophagy to remove damaged mitochondria while targeting ubiquitinated NLRP3 for degradation, thereby inhibiting inflammasome assembly. By disrupting the vicious cycle of NLRP3 activation and mitochondrial dysfunction, Nrf2 establishes a neuroprotective barrier against synergistic inflammatory and oxidative insults (Rajan et al., 2023; Figure 3).

#### 2.2.4 Reduction of iron deposition

Ferroptosis has emerged as a critical pathogenic mechanism in neurodegenerative diseases (Zhang et al., 2021). In PD, the substantia nigra exhibits markedly elevated free iron concentrations alongside reduced iron-storing neuromelanin, leading to increased cytoplasmic iron release (Shamoto-Nagai et al., 2006). PD models consistently reveal disrupted iron homeostasis, characterized by upregulation of divalent metal transporter 1 (DMT1) and downregulation of ferroportin 1, thereby promoting excessive cellular iron accumulation (Zhang et al., 2021). Concurrently, total brain ferritin levels are decreased despite elevated ferritin deposition in the substantia nigra, where an increased ferritin heavy chain-to-light chain ratio is associated with reduced iron storage stability (Friedman and Galazka-Friedman, 2012).

The Fenton reaction, in which ferrous ions catalyze the conversion of H_2_O_2_ into highly reactive hydroxyl radicals (•OH), initiates lipid peroxidation by abstracting hydrogen from polyunsaturated fatty acids, ultimately driving ferroptotic cell death (Dixon et al., 2012). In PD, the lipid peroxidation byproduct 4-hydroxy-2,3-trans-nonenal (4-HNE) promotes pathological protein aggregation, including α-Syn oligomerization, and induces mitochondrial dysfunction through protein adduct formation (Bae et al., 2013; Gow et al., 1996). These modifications exacerbate electron transport chain leakage and amplify ROS generation (Lee and Hyun, 2023). The ensuing oxidative stress activates NF-κB signaling, stimulates neuroinflammatory glial responses, and promotes P2X purinoceptor 7 (P2X7R)-mediated inflammasome assembly, thereby establishing a self-perpetuating cycle of neurodegeneration (Lee and Hyun, 2023; Mattson and Camandola, 2001).

Experimental studies in islet transplantation and thrombosis models demonstrate that BL exerts anti-ferroptotic effects through multiple mechanisms, including upregulation of HO-1/Nrf2 signaling, enhancement of cellular viability, elevation of antioxidant capacity, and reduction of cell death (Yao et al., 2020a,b). Manikanta et al., 2024, the potent Fe^2+^ ion–chelating capacity of BL effectively attenuates intracellular iron accumulation (Xing et al., 2019). Bilirubin can alleviate hepatic oxidative stress, maintain normal hepcidin function, and upregulate GPX4 to prevent iron overload (Shinn et al., 2024; Yao et al., 2020a,b). Moreover, Nrf2—a key regulator of iron homeostasis—is robustly activated by BL (Yao et al., 2020a,b), leading to increased production of NAD(P)H-dependent enzymes such as NQO1, suppression of NF-κB signaling, and reduction of both α-Syn aggregation and oxidative stress–mediated neuroinflammatory damage (Lastres-Becker et al., 2016; Liu et al., 2017; Sivandzade et al., 2019). *In vitro* studies further indicate that biliverdin exhibits moderate ferroptosis-inhibitory activity (Nakajima et al., 2024). However, the potential neuroprotective role of BL in modulating ferroptosis within the nervous system remains to be conclusively established and warrants further investigation.

### 2.3 Neonatal jaundice may reduce risk of PD development

#### 2.3.1 Mechanisms underlying early development of DA neurons

During mammalian embryonic development, Ventral Tegmental Area (VTA) DA neurons arise from the midbrain floor plate, where radial glial-like neural progenitors differentiate into three functionally distinct populations (Garritsen et al., 2023; Ono et al., 2007): the A8 retrorubral field, A9 substantia nigra pars compacta (SNc), and A10 ventral tegmental area (Toulouse and Sullivan, 2008). Developmental studies indicate that floor plate–derived Shh signaling, initiated around embryonic day 8.5 in mice, orchestrates the sequential generation of VTA DA neurons, with ventral tegmental area neurons emerging before SNc neurons (Echelard et al., 1993; Ho and Scott, 2002). Concurrently, Pax2-driven induction of fibroblast growth factor 8 (FGF8) expression in the isthmic organizer establishes the molecular framework for VM DA progenitor patterning (Ye et al., 2001). This FGF8 signaling cascade subsequently activates Wnt1 expression in the midbrain, which in turn upregulates the Engrailed-1/2 transcription factors—first detected at embryonic days 11.5–14 in mice—whereupon their sustained expression becomes essential for the maintenance and survival of VTA DA neurons (Hegarty et al., 2013).

*In vitro* experiments reveal the critical involvement of Wnt signaling in embryogenesis and organ development. Wnt1 stimulates VTA DA neural progenitor proliferation and boosts DA neuron generation, while Wnt5a supports DA phenotypic determination (Castelo-Branco et al., 2003, 2006). The canonical Wnt/β-catenin pathway also modulates Shh signaling, thereby alleviating its inhibitory effects on VTA DA neural progenitors proliferation during later developmental stages (Joksimovic et al., 2009a,b; Tang et al., 2010). Double mutant embryos exhibit normal initial development of VTA DA neurons, followed by complete caspase-dependent apoptotic loss by embryonic day 14 (Albéri et al., 2004).

During VTA DA neuron differentiation, Lmx1a and Lmx1b are co-expressed in VTA DA neural progenitors and function as critical mediators of early DA specification (Andersson et al., 2006; Chakrabarty et al., 2012; Deng et al., 2011; Zou et al., 2009). These transcription factors also contribute to VTA DA neuron maturation; ectopic expression of Lmx1a and Lmx1b can induce VTA DA neuron generation in ectopic locations, whereas loss of Lmx1b markedly reduces the VTA DA neuron population (Deng et al., 2011; Nakatani et al., 2010). Concurrently, the transcription factor FoxA2 is both necessary and sufficient for floor plate development, directing floor plate neural progenitors toward a DA fate and playing a pivotal role in DA phenotype induction (Ang and Rossant, 1994; Hegarty et al., 2013; Nakatani et al., 2010). FoxA2 mutant mice exhibit defects in both floor plate and notochord development, resulting in embryonic lethality by embryonic day 9.5 (Ang and Rossant, 1994). Furthermore, FoxA2 and FoxA1 sustain Lmx1a and Lmx1b expression, thereby reinforcing VTA DA neurogenesis (Bayly et al., 2012; Ferri et al., 2007; Lin et al., 2009).

The nuclear receptor transcription factor Nurr1 orchestrates DA neuron maturation, functional regulation, and lifelong maintenance, with its expression—initiating in the VM at embryonic day 10.5 (in mice) and persisting throughout adulthood—mediating perinatal stress response adaptation (Zetterström et al., 1996). The Wnt/β-catenin pathway enhances regulatory effects of Nurr1, including TH promoter activation, indicating that Nurr1 is a key determinant of VTA DA neurotransmitter identity by controlling genes involved in DA synthesis, vesicular packaging, axonal transport, and reuptake (Arenas, 2005; Jankovic et al., 2005). Nurr1 is also critical for the long-term survival of VTA DA neurons (Kadkhodaei et al., 2009). Pitx3, although not required for the early development or migration of VTA DA neurons (Smidt et al., 1997), begins expression at embryonic day 11.5 and is essential for SNc neuron specification. In Pitx3-deficient mice, SNc DA neurons fail to express TH, leading to loss of nigrostriatal projections (Hwang et al., 2003; Smidt et al., 2004), whereas TH expression in ventral tegmental area neurons remains unaffected (Korotkova et al., 2005). Evidence suggests that Nurr1 and Pitx3 cooperate to regulate the expression of DA neurotrophic factors such as brain-derived neurotrophic factor and glial cell line-derived neurotrophic factor, as well as genes critical for DA neurotransmission, thereby promoting VTA DA neuron survival and the acquisition of mature DA phenotypes (Chakrabarty et al., 2012; Hegarty et al., 2013; Hwang et al., 2009; Martinat et al., 2006; Table 1).

**Table 1:** An essential molecular regulator of dopaminergic neuron development.

**Target**	**Timing of action in embryonic development**	**Impact on neural development**	**References**
Shh	Around embryonic day 8.5 in mice	Promotes VM DA neuron generation and differentiation, sequentially producing VTA DA and SNc DA	Hegarty et al. (2013); Schwamborn (2018)
FGF8	Early patterning stage	Induces proper patterning of VM DA NPs and promotes midbrain Wnt1 expression	Bolam and Pissadaki (2012); Xing et al. (2019)
Wnt-β-catenin	Early proliferation/differentiation and mid-late stages	Early: increases DA neuron numbers and promotes differentiation; Late: suppresses Shh levels, reducing its inhibition on VM DA NP proliferation; Enhances Nurr1-mediated gene regulation	Ho and Scott (2002); Echelard et al. (1993); Shinn et al. (2024); Yao et al. (2020a,b); Lastres-Becker et al. (2016)
Lmx1a/Lmx1b	Early specification stage	Mediates initial steps of VM DA NP specification and promotes DA neuron maturation; loss causes severe reduction in VM DA neurons	Sivandzade et al. (2019); Nakajima et al. (2024); Garritsen et al. (2023); Ono et al. (2007)
Nurr1	embryonic day 10.5 to adulthood	Master regulator of DA neuron maturation/survival; controls DA synthesis/transport genes; cooperates with Pitx3 to regulate BDNF/GDNF. Wnt/β-catenin enhances TH activation.	Castelo-Branco et al. (2003); Albéri et al. (2004); Chakrabarty et al. (2012); Deng et al. (2011)

Early-life LPS exposure induce microglial activation and triggers persistent DA neuron loss from infancy through adulthood, with maximal vulnerability observed at embryonic day 10.5—a critical developmental window for DA neurogenesis (Arenas, 2005; Block and Hong, 2005; Zetterström et al., 1996).

#### 2.3.2 Mechanisms underlying potential protective mechanisms of neonatal jaundice on DA neuron development

Emerging evidence suggests that neonatal jaundice may provide neuroprotective effects against PD by supporting proper DA neuron development (Falcão et al., 2013; Hansen et al., 2020; Llido et al., 2023). The Wnt signaling pathway, essential for early DA neurogenesis, has been implicated in increased neurodegenerative disease risk when dysregulated (Catlin and Schaner Tooley, 2024; Wang et al., 2020). Experimental studies demonstrate that BRNPs significantly upregulate Wnt pathway activity in myocardial ischemia-reperfusion models (Kim et al., 2024), whereas research in colon cancer shows that biliverdin reductase A-mediated BL production modulates Wnt/β-catenin signaling through bidirectional regulation of its target genes (Mao et al., 2020). During DA neurogenesis, BL appears to enhance neuronal proliferation via Wnt pathway activation and promote TH-positive neuron maturation and long-term survival through Wnt/β-catenin-dependent Nurr1 expression.

Pharmacological studies suggest coordinated regulation of FGF, Wnt, and Shh signaling with serum BL levels (Hayashida et al., 2020; Tao et al., 2022; Zhang Y. et al., 2018), a relationship further supported by our unpublished Mendelian randomization analyses indicating neonatal jaundice as a potential protective factor against PD onset. Additionally, HO-1-derived BL and CO have been shown to upregulate brain-derived neurotrophic factor and glial cell line-derived neurotrophic factor expression in both neurons and astrocytes, thereby facilitating proper DA neuron development (Hung et al., 2010).

## 3 Discussion

Bilirubin, as an endogenous metabolite, is gaining increasing attention for its multi-target protective effects in PD. This study synthesizes current evidence to demonstrate that physiological or mildly elevated levels of UCB may protect DA neurons through multiple mechanisms, offering new insights for PD prevention, and treatment.

Firstly, bilirubin exhibits multi-faceted regulatory capabilities targeting the core pathological processes of PD. Regarding oxidative stress, bilirubin directly scavenges reactive species such as ROS and NO, protects mitochondrial electron transport chain function, and maintains stable energy metabolism in DA neurons (Cui et al., 2023). This effect is particularly important given the significant mitochondrial dysfunction characteristic of PD pathology. In terms of neuroinflammation regulation, bilirubin not only increases the proportion of M2 microglia to promote an anti-inflammatory environment but also modulates peripheral immune cell infiltration by reducing CD4+ T cell aggregation, increasing Treg cell proportion, and inhibiting the production of pro-inflammatory factors like TNF-α, thereby breaking the vicious cycle of neuroinflammation (Idelman et al., 2015). More notably, bilirubin's regulation of ferroptosis—by alleviating iron deposition and activating the Nrf-2 pathway—enhances the cellular antioxidant defense system and reduces programmed death of DA neurons under stress conditions (Dinkova-Kostova and Abramov, 2015).

Secondly, this study proposes a forward-looking perspective: bilirubin's protective effects may extend to the developmental stage of DA neurons during embryogenesis. Growing evidence suggests that abnormal neural development is a potential early factor in neurodegenerative diseases including PD. Bilirubin may influence the differentiation, migration, and survival of DA neurons by modulating key developmental pathways such as Wnt/β-catenin, Shh, and FGF, while promoting the secretion of neurotrophic factors. Such early intervention could fundamentally enhance the stress resistance of DA neurons and reduce an individual's long-term susceptibility to PD (Hwang et al., 2009; Martinat et al., 2006).

Based on these mechanisms, bilirubin-related therapies demonstrate unique therapeutic potential. Unlike current PD treatments that primarily alleviate symptoms, bilirubin's multi-target nature may allow simultaneous intervention in multiple disease progression pathways, achieving “multi-efficacy with a single agent.” However, translating bilirubin into clinical therapy still faces challenges: determining the optimal treatment window, selecting administration routes (especially efficient blood-brain barrier penetration), evaluating long-term safety, and establishing individualized dosing regimens all require in-depth research. Particularly, how to precisely maintain its protective concentration in the central nervous system without inducing bilirubin neurotoxicity is a core issue for future studies.

Based on these mechanisms, bilirubin demonstrates unique therapeutic potential, offering distinct advantages over established PD treatments. Unlike the gold-standard Levodopa, which primarily alleviates symptoms but paradoxically generates iatrogenic ROS through auto-oxidation (Scigliano et al., 1997), bilirubin acts as an endogenous disease-modifying agent that specifically neutralizes such oxidative burdens (Jayanti et al., 2021). Furthermore, compared to consumed antioxidants (e.g., Vitamin E) or single-target iron chelators (e.g., Deferiprone), bilirubin is continuously regenerated via the highly efficient biliverdin-bilirubin redox cycle. This enables a distinct multi-targets advantage, simultaneously chelating iron, inhibiting lipid peroxidation, and dampening neuroinflammation, thereby achieving multi-efficacy with a single agent (Jayanti et al., 2023). However, translating native bilirubin into clinical therapy faces pharmacological challenges. Primarily, its poor aqueous solubility, chemical instability, and narrow therapeutic window where supraphysiological levels may induce neurotoxicity (Cayabyab and Ramanathan, 2019; Mancuso, 2017). Therefore, developing advanced drug delivery systems, such as bilirubin nanomedicines, represents a critical direction for the future clinical translation of bilirubin-based therapies (Lee et al., 2016).

In summary, bilirubin provides a new biological perspective for PD prevention and treatment through multiple mechanisms, including antioxidant, anti-inflammatory, anti-ferroptosis effects, and potential neural development regulation. These findings not only deepen our understanding of PD pathogenesis but, more importantly, suggest a feasible strategy for disease-modifying therapy utilizing endogenous protective substances. Future research should focus on elucidating the precise network of bilirubin's actions in the central nervous system, exploring its synergistic effects with other PD treatments, and advancing critical steps toward clinical translation, ultimately offering more fundamental therapeutic options for PD patients.
